# Proliferative cell nuclear antigen (PCNA) expression in the intestine of *Salmo trutta trutta* naturally infected with an acanthocephalan

**DOI:** 10.1186/1756-3305-5-198

**Published:** 2012-09-11

**Authors:** Bahram Sayyaf Dezfuli, Luisa Giari, Alice Lui, Samantha Squerzanti, Giuseppe Castaldelli, Andrew Paul Shinn, Maurizio Manera, Massimo Lorenzoni

**Affiliations:** 1 Department of Biology & Evolution, University of Ferrara, St. Borsari 46, 44123, Ferrara, Italy; 2 Institute of Aquaculture, University of Stirling, Stirling, Scotland, FK9 4LA, UK; 3 Department of Food Science, University of Teramo, St. Crispi 212, 64100, Teramo, Italy; 4 Department of Cellular and Environmental Biology, University of Perugia, St. Elce di Sotto 5, 06123, Perugia, Italy

**Keywords:** Cell proliferation, Immunohistochemistry, Fish intestine, Enteric helminth.

## Abstract

**Background:**

Changes in the production of proliferating cell nuclear antigen (PCNA), a 36 kd protein involved in protein synthesis, within intestinal epithelia can provide an early indication of deviations to normal functioning. Inhibition or stimulation of cell proliferation and PCNA can be determined through immunohistochemical staining of intestinal tissue. Changes in the expression of PCNA act as an early warning system of changes to the gut and this application has not been applied to the fields of aquatic parasitology and fish health. The current study set out to determine whether a population of wild brown trout, *Salmo trutta trutta* (L.) harbouring an infection of the acanthocephalan *Dentitruncus truttae* Sinzar, 1955 collected from Lake Piediluco in Central Italy also effected changes in the expression of PCNA.

**Methods:**

A total of 29 brown trout were investigated, 19 of which (*i.e.* 65.5%) were found to harbour acanthocephalans (5–320 worms fish^-1^). Histological sections of both uninfected and infected intestinal material were immunostained for PCNA.

**Results:**

The expression of PCNA was observed in the epithelial cells in the intestinal crypts and within the mast cells and fibroblasts in the submucosa layer which is consistent with its role in cell proliferation and DNA synthesis. The number of PCNA-positive cells in both the intestinal epithelium and the submucosa layer in regions close to the point of parasite attachment were significantly higher than the number observed in uninfected individuals and in infected individuals in zones at least 0.7 cm from the point of parasite attachment (ANOVA, *p *< 0.05).

**Conclusions:**

An infection of the acanthocephalan *D. truttae* within the intestinal tract of *S. t. trutta* effected a significant increase in the number of PCNA positive cells (mast cells and fibroblasts) at the site of parasite attachment when compared to the number of positive cells found in uninfected conspecifics and in tissue zones away from the point of parasite attachment.

## Background

Changes in the rate of normal cell proliferation within the intestinal tract may serve as an early indication of abnormality. These changes are frequently screened in toxicity bioassays [1]. The intestinal epithelium undergoes rapid cell turnover and this renewal relies on intestinal stem cells situated in the crypt of the finger-like intestinal villi to generate new cells which consequentially migrate along the axis of the villi [2]. Deregulation of intestinal cell proliferation and differentiation impairs the renewal of the intestinal epithelium that underlies many digestive diseases [2]. Cell proliferation can be detected by immunohistochemical staining of the proliferating cell nuclear antigen (PCNA) (see [3]), which is an evolutionary, highly conserved 36 kd protein that is directly involved in DNA synthesis [4]. PCNA immuno-positivity is confined largely to the nuclei of dividing sperm cells, in ovarian follicles, lymphoid tissues of the kidneys, in neuronal cells of proliferative regions of the brain, epithelial cells of gill filaments, and, in the proliferative zones in the crypt regions of mucosal folds [5-7]. In teleosts, the presence of these proliferating cells at the base of intestinal folds have been described in *Ctenopharyngodon idella* (Valenciennes) [8] and in *Barbus conchonius* (F. Hamilton) [9].

In all vertebrates, the alimentary canal represents one of the primary routes of parasite and pathogen infection [10], and, therefore, serves as the primary barrier limiting or preventing the entry of harmful agents [11]. Under normal conditions, healthy fish are able to defend themselves against a broad spectrum of pathogens using a complex system of innate defence mechanisms [12]. The alimentary canal has a series of well-developed chemical and physical barriers which cooperate with an efficient, local, mucosal immune system [13]. Helminths comprise a diverse group of metazoan organisms consisting of several phyla and class of parasite presenting a vast array of differing morphologies, feeding strategies, behaviours and life-cycles [14,15], each exploiting a different niche within the intestinal environment. In a trade-off between host and parasite, many intestinal helminths have evolved mechanisms to evade their host’s immune response, whilst the hosts have evolved a series of counter measures to deal with these [16]. As part of the infection process, certain intestinal helminths may induce structural modification to their host’s tissues, and provoke alterations to the normal intestinal physiology [17]. In fish, the innate defences responding to helminth infection are associated with an inflammatory reaction [10,18]. Enteric helminths elicit an increase in the migration and accumulation of certain types of host immune cell (*e.g.*, granulocytes) at the site of infection [18-20]. A histopathological approach paying particular attention to the cellular response initiated by the host in response to a helminth infection, which when combined with immunohistochemical methods, will provide a basis for the future elucidation on piscine antihelminthic responses [21].

This study examines changes in the number of PCNA-positive cells in the intestine of a teleost fish infected with an enteric parasite. Specifically, the study looks at the number of proliferating cells seen in the immediate vicinity of an acanthocephalan attaching to the intestine of a brown trout, *Salmo trutta trutta* (L.), and compares them to the number observed away from the point of parasite attachment and in uninfected conspecifics.

## Results

**Figure 1:**
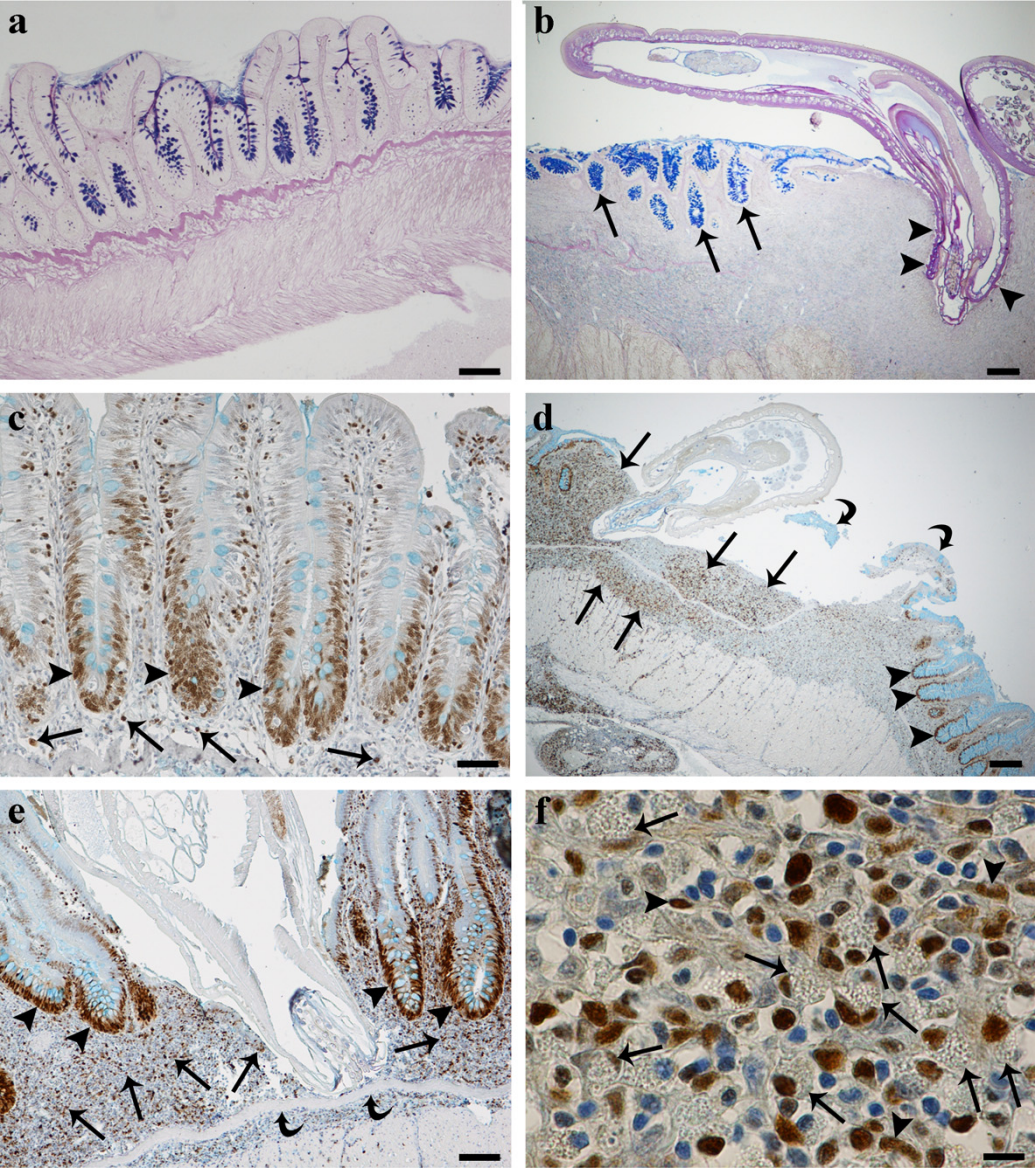
**Histological sections through the intestines of *Salmo trutta trutta* L.** (**a**) An H&E stained section through an uninfected intestine where intact villi can be seen, scale bar = 100 μm. (**b**) Haematoxylin and eosin stained section of a trout intestine with an attached *Dentitruncus truttae in situ*. Note the spines on the trunk of the acanthocephalan (arrow heads) and the destruction of the villi at the site of attachment whilst those further away (arrows) are still intact, scale bar = 200 μm (**c**-**f**) Immunohistochemistry. (**c**) Section through an uninfected intestine that has been stained with a PCNA-antibody. Positive cells (arrow heads) are localised deep within the intestinal folds. A small number of positive cells (arrows) are also visible within the sub-mucosal layer, scale bar = 50 μm. (d) Section through an infected brown trout intestine that has been stained with the PCNA-antibody. There is a lack of villi at the site of *D. truttae* attachment and numerous positive cells (arrows) within the sub-mucosal layer are evident. Note the residual pieces of damaged intestinal fold (arrow heads). Numerous immunoreactive epithelial cells (curved arrows), however, are visible deep within the intestinal folds further away from the site of parasite attachment, scale bar = 200 μm. (**e**) An acanthocephalan with a proboscis which has not penetrated the *stratum granulosum* (curved arrow). Numerous PCNA-positive epithelial cells (arrow heads) are evident deep within the villi. Note the immunoreactive cells (arrows) within the sub-mucosal layer, scale bar =50 μm. (**f**) A higher magnification of the *lamina propria*-*stratum granulosum* at a point in close proximity to the acanthocephalan proboscis where numerous PCNA-positive mast cells (arrows) and some positive fibroblasts (arrow heads) are evident, scale bar = 10 μm.

Of the 29 specimens of *S. t. trutta* (32.27 ± 5.71 cm, mean total length ± standard deviation [S.D.]) collected from Lake Piediluco, ten were uninfected and from the histological sections that were taken, the architectural integrity of the intestinal folds appeared to be intact (see Figure 1a). The remaining 19 (65.5%) brown trout were infected with the acanthocephalan *Dentitruncus truttae* Sinzar, 1955 (Figure 1b) with the intensity of infection ranging from 5 to 320 worms per fish. The majority of worms were found in the median intestine. Generally, the acanthocephalans did not penetrate the *stratum granulosum* (Figures 1,b, d e), although occasionally a proboscis was observed to have penetrated the deeper layers causing disruption to the mucosa, *lamina propria*, *stratum granulosum* and the *muscularis* layers at the point of proboscis insertion (not shown). *Dentitruncus truttae,* with its numerous trunk spines, were observed in contact with the intestinal epithelium where they caused damage to the apices of villi (Figures 1,b, d e). The spines caused detachment of the epithelial cells and reduced the number of mucosal folds (Figure 1d). By comparison, the intestinal folds at sites at a distance of 0.7 cm or greater from the point of parasite attachment, remained intact (Figures 1b, d).

**Figure 2:**
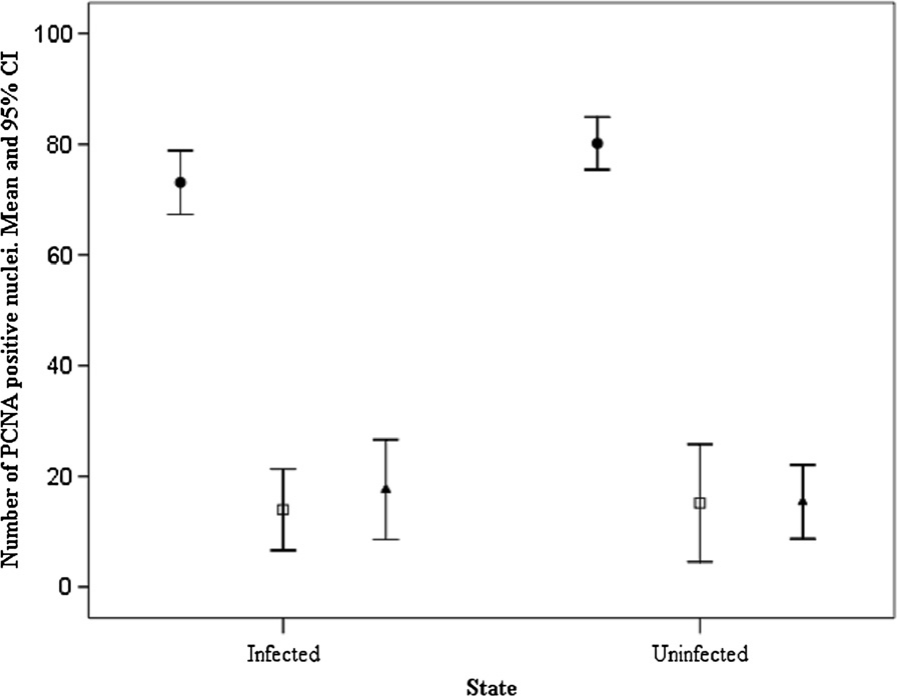
**The mean number of PCNA positive nuclei in the intestines of uninfected *Salmo trutta trutta* and in infected hosts at a distance from the point of parasite attachment.** Error bars represent 95% confidence intervals (CI) about the mean. Abbreviations: ●, epithelial cells; □, mast cells; ▴, fibroblasts.

**Figure 3:**
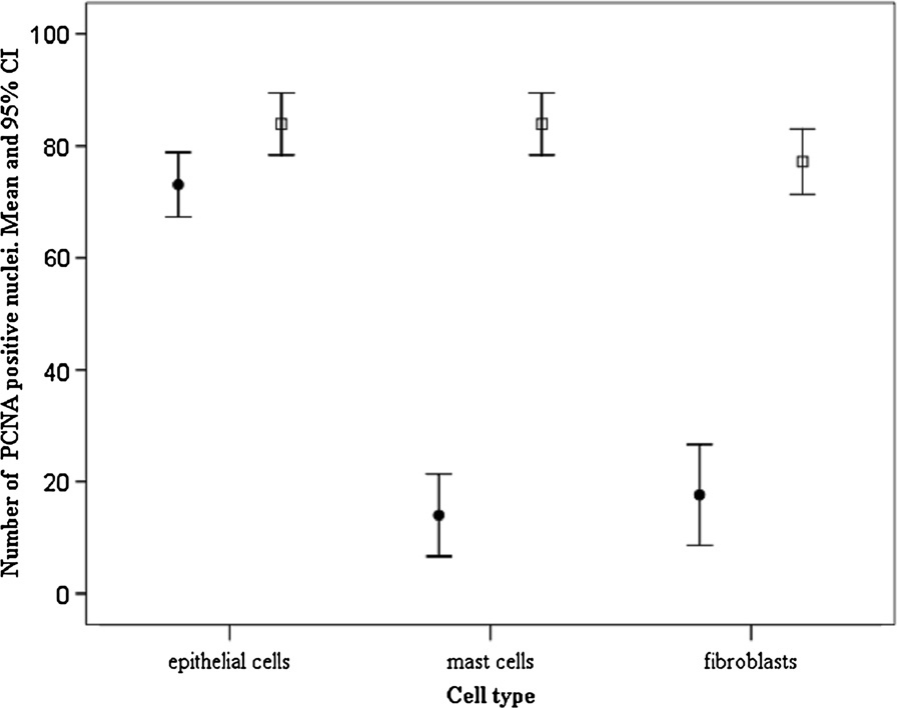
**The mean number of PCNA positive nuclei by cell type (*i.e. *epithelial cell, fibroblast or mast cells) in the intestines of infected *Salmo trutta trutta * at the site of parasite attachment and at points further away.** Error bars represent 95% confidence intervals (CI) about the mean. Abbreviations: ●, sites at a distance from the point of parasite attachment; □, sites close to the point of parasite attachment.

The intestinal mucosa of *S. t. trutta* is lined by a simple epithelium consisting of typical columnar epithelial cells with a sparse intermingling of mucous cells (Figure 1c). The response pattern for PCNA-positivity along the intestinal folds was common in the basal area of folds in both uninfected (Figure 1c) and acanthocephalan infected *S. t. trutta* (Figures 1d, e). Data from the immunohistochemical tests with the PCNA antiserum and positive cell counts are presented graphically in Figures 2 and 3.

No significant differences were observed in the number of PCNA positive nuclei in the intestinal sections taken from uninfected fish and from the infected trout but at a distance from the point of parasite attachment (ANOVA, *p *> 0.05) (Figure 2). In infected fish, the number of PCNA-positive cells in the intestinal epithelium and/or the submucosa layer close to the site of parasite attachment (Figures 1d, e) were significantly higher than the numbers observed at points situated 0.7 cm or greater away from the point of parasite attachment (ANOVA, *p *< 0.05) (Figure 3) and from uninfected individuals (ANOVA, *p *< 0.05). Numerous PCNA-positive cells, for example, were observed in the *lamina propria-stratum granulosum* in the immediate vicinity around the proboscis of *D. truttae* (Figures 1d, e). Parallel studies with light and electron microscopy have shown that these positive cells are mast cells (MCs; Figure 1f) – these were the only positive type of leucocyte found. These cells are typically oval in shape, with an eccentric, polar nucleus, and a cytoplasm characterised by numerous large, membrane-bounded granules. Numerous MCs were seen within the *stratum granulosum* and in the *muscularis* layer, in close contact with capillaries. MCs were also observed in the outer layer of the endothelia, as well as inside blood vessels (not shown). MCs were frequently surrounded by fibroblasts and collagen fibres. Some fibroblasts were PCNA positive (Figure 1f), although it is interesting to note that positivity of these and MCs was influenced by their distance from the parasite (Figure 3). In infected fish, each cell type, and notably for the MCs and fibroblasts, the number of positive nuclei increased significantly (ANOVA, *p *< 0.01) at the site of parasite attachment (Figure 3). In this study, the epithelial cells displayed higher PCNA positivity than the MCs and fibroblasts (ANOVA, *p *< 0.01; Figure 2). There were no statistical differences in the number of MCs and fibroblasts (ANOVA, *p *> 0.05; Figure 2).

## Discussion

The PCNA is a ring-like protein that provides the DNA polymerase the processivity for DNA replication [22]. It is believed that the gene sequence and functions of PCNA are remarkably conserved among eukaryotes [23]. PCNA has been reported in several cell types in mammalian tissues and PCNA-positivity has been reported from a number of different organs in fish [2,3,24,25]. The aquatic environment is continuously exposed to a range of organic chemicals and there is growing concern in how each of these compounds affects the molecular and cellular mechanisms within the intestinal tract [26]. An increase in expression of PCNA is widely accepted as a marker of proliferation associated with the development of neoplastic tissue [3,24,27,28]. Teleost fish have, therefore, become a popular model for use in cancer studies, where there is growing interest on the concurrent detection of PCNA, tumour protein p53 and apoptosis [23,29,30].

The stimulation or inhibition of normal cell proliferation, therefore, serves as an early indication of potential abnormality within the intestinal tract, making these an appropriate model for study in toxicity bioassays [1,31]. New intestinal epithelial cells are continuously produced by stem cells in the crypts which subsequently migrate along the crypt-villi axis [26]. An increase in PCNA labelling, therefore, signals marked increases in the rate of cellular division. A number of experimental studies have examined the cellular localisation of PCNA within the intestines of fish exposed to a model toxicant [31] and through the dietary administration of compounds [25,32,33], but no information exists regarding the expression of PCNA in infected fish tissues. The acanthocephalan *D. truttae,* in addition to its armed proboscis, bears trunk spines that facilitate its attachment within the intestinal villi of its fish host [34]. These spines, during the process of attachment, inflict damage to the intestinal folds, causing destruction of the villi epithelium resulting in the development of necrotic tissue. The immunohistochemical results demonstrate that the levels of acanthocephalan infection observed in the current study (*i.e.* 5–320 individuals fish^-1^) effect a significant increase in the number of PCNA-positive cells in the intestinal villi that are close to the sites of parasite attachment. The increase in PCNA-positive epithelial cells were observed within the villi crypts that were either occupied by or close to intestinal helminths, when compared to the lower numbers seen in uninfected fish or in villi that were situated at least 0.7 cm away from the site of helminth activity.

The extent of intestinal damage inflicted by acanthocephalans is related to the intensity of infection and the degree to which the parasite penetrate the host tissues [35,36]. Parasitic infection of the alimentary canal can have detrimental effects on digestive function [10,37], with many species of intestinal helminth inducing an inflammatory response at the site of attachment [21,38]. In fish, the innate defences in response to helminth infection are associated with inflammatory reactions [10,18,39]. The innate immunity of teleosts, involves a range of cell types, which commonly include MCs [13,40,41]. In perciform fish, it has been reported that the mast cells contain histamine, which can regulate the fish’s inflammatory responses [42]. Moreover, MCs degranulation has been shown to promote intestinal contraction in *Sparus aurata* L. and in *Oncorhynchus mykiss* (Walbaum) [42,43].

Although Roberts et al. [44] introduced the term eosinophilic granule cell, there has been a tendency in recent years to use the term mast cell as these cells have functional and morphological similarities to [mammalian] MCs [45,46]. MCs are present in most species of teleost and are found in a variety of tissues, including the gastrointestinal tract, skin and gills [45-47]. MCs are motile [19,48] and until recently, their origin was unclear but it is now known that in mammals their precursors are from pluripotent bone marrow-derived stem cells circulating in the blood and lymphatic fluid [49]. In fish, it is most likely that MCs differentiate in the haematopoietic organs and reach their target tissues via the circulatory system as immature cells [50], as has been reported for mammalian MCs [51].

Evidence of MCs migration has been documented in the gills of rainbow trout, *O. mykiss*, exposed to bacteria [52] and in the intestine of fish infected with tapeworms [53]. In all vertebrates, MCs may be strategically positioned at perivascular sites to regulate inflammatory responses [42,49]. This places them in a unique position to encounter invading organisms and to orchestrate a response [49]. In the current study, and in particular within the intestines of infected fish, numerous MCs were seen in close contact with capillaries and the outer layer of the endothelia as well as within the lumen of the blood vessels. MCs play an important role in responding to inflammation; their number increases in allergic reactions [49] and as a consequence of helminth infection [39,53-55]. The close association of MCs with the endothelial cells of capillaries and their presence within gill capillaries suggests that they may migrate across the endothelium [47,52,54]. Nonetheless, the intra-tissue migratory nature of MCs has been observed in the gills and intestine of fish [48,56,57]. In addition, the occurrence of MCs throughout the loose connective tissue of the gill arch, suggests that there is a resident population of these cells [47,56]. In the current study, numerous MCs were observed within the connective tissue, and both on the outside and within capillaries in the sub-mucosal layers of infected brown trout intestines. Based on a considerable body of descriptive data, it is reasonable to presume that fish may have two populations of MCs, a circulating and a resident population, and the presence of parasites may induce recruitment of MCs to the site(s) of infection [18,20]. Accordingly, acute MC activation is a feature of many types of tissue injury; experimental studies have demonstrated that pathogen products can activate MCs [58].

In humans, PCNA-positive MCs have been observed in the nasal sub-epithelial and *lamina propria* layers in patients suffering allergic rhinitis [59]. A proliferation of mature MCs has also been reported in the nasal mucosa of those suffering allergies [60-62]. In the current study, a high number of PCNA-positive cells were seen in the sub-mucosal layer, immediately around the proboscis of *D. truttae*; most of these were MCs with some fibroblasts. The co-occurrence of fibres-fibroblasts and MCs has been described from a range of fish species including *O. mykiss* (see [50] Flaño et al. 1996), coho salmon, *Oncorhynchus kisutch* (Walbaum) (see [63]) and minnows, *Phoxinus phoxinus* L. (see [64]). It has been suggested that MCs have the potential to directly influence fibroblasts and/or indirectly influence other cells, leading to a profibrotic response [65]. Several lines of evidence suggest that MCs are involved in the fibrotic process and in tissue remodelling [66,67]. In the process of attachment, the proboscis of *D. truttae* penetrates deep into the sub-mucosal layer and destroys the architecture of the host’s intestinal wall; the recruitment and proliferation of numerous MCs around the site of proboscis insertion may serve also to repair and remodel damaged intestinal tissue.

## Materials and methods

During 2011, a total of 29 specimens of brown trout, *S. t. trutta*, were processed from Lake Piediluco (Province of Terni, Central Italy; 42° 31´ 01" N; 12° 45´ 00" E). The fish were caught by gill net that was deployed on two occasions by professional fishermen belonging to the Piediluco Fish Consortium. Immediately on landing, the fish were removed and transferred alive to the Consortium’s facility where they were euthanased using an overdose of 125 mg L^-1^ MS222 (tricaine methanesulfonate, Sandoz, Basel, Switzerland). Thereafter, the spinal cord was severed and the fish lengthed and weighed. On post mortem, the fish were sexed before the digestive tract was removed and opened longitudinally in search of helminths. For parasites found still attached to the intestine, their exact position was recorded before a 15 × 15 mm piece of tissue that surrounded the site of attachment was excised and then fixed in Bouin’s for 10 h. Thereafter, the fixed pieces of tissue were rinsed in several changes of 4°C 70% ethanol before being stored in the same medium until they were processed for histology. After fixation, the tissues were dehydrated through an alcohol series and then paraffin wax embedded using a Shandon Citadel 2000 Tissue Processor. After blocking out, 5 μm thick sections were taken from each tissue block and the slides dried at 60°C for several hours. After dewaxing in xylene and rehydrated through a graded alcohol series, the slides were treated for antigen retrieval in citrate buffer pH 8.0 for 20 min in a steamer bath at 95°C; thereafter the slides were left for 10 min to cool to room temperature (RT). Endogenous peroxidase activity and non-specific staining were blocked respectively in 3% H_2_O_2_ for 10 min and then in horse normal serum (1:20) for 30 min. Commercially available antibody anti-PCNA (PC10 sc-56 mouse anti-rat IgG2a monoclonal antibody, Santa Cruz Biotechonology, Inc.), recommended for detection of PCNA in cells in a broad range of organisms including mammals, insects and yeasts, was used. Sections were incubated with the primary antibody (anti-PCNA diluted 1:500) for 2 h at RT. After washing with PBS, slides were incubated for 30 min with biotinylated horse anti-mouse serum (Vector, Burlingame, USA) followed by avidin-conjugated horseradish peroxidase (Vector, Burlingame, USA). The enzyme activity was detected using DAB (3,3’-diaminobenzidine). Non immune mouse serum and diluent only sections were used as negative controls. Finally, the sections were dehydrated, counterstained with alcian blue and Harris' haematoxylin, mounted in Canada balsam, examined and photographed using a Nikon Microscope ECLIPSE 80i.

For comparative purposes, intestinal sections from ten acanthocephalan infected and ten uninfected trout were screened for PCNA positive cells. For each trout and each cell type (*i.e.* epithelial cells, mast cells and fibroblasts), 100 nuclei were assessed at × 400 magnification and the ratio of PCNA positive nuclei were determined. In infected specimens, a nuclear count was made at the point of parasite attachment with a second count made 0.7 cm away; this approach allowed for differences in PCNA nuclear positivity due to helminth infection within the same host to be considered. Data, the number of PCNA positive nuclei, were assessed for normality by means of the Kolmogorov-Smirnov and Shapiro-Wilk tests. Parametric tests were subsequently applied and a generalised linear model for repeated measures was applied. Paired measures of PCNA positive cells in infected fish (at the site of infection and at a distance from it) were introduced in the model as repeated measures, whereas cell types (epithelial cells, mast cells and fibroblasts) were introduced in the model as fixed factors. Thus, differences between sites (at the site of attachment and at a distance from it), cell types (epithelial cells, mast cells, fibroblasts) and their interactions were evaluated. Moreover, a one way ANOVA was performed to detect significant differences in the number of positive cells determined from the uninfected and infected intestines (away from site of parasite attachment). The statistical package SPSS 14.0.2 and a *p* < 0.05 level of significance were used throughout.

## Conclusions

Changes in the production of PCNA within intestinal epithelia can provide an early indication of deviations to normal functioning. In brown trout, the number of PCNA-positive cells in both the intestinal epithelium and the submucosa layer in regions close to the point of *Dentitruncus truttae* attachment were significantly higher than the number observed in uninfected individuals and in infected individuals in zones away from the point of parasite attachment. The study also demonstrates the presence of PCNA positive mast cells and fibroblasts within the intestinal tissues of a fish.

## Abbreviations

CI: Confidence intervals; DAB: Diaminobenzidine; MCs: Mast cells; PBS: Phosphate buffered saline; PCNA: Proliferating cell nuclear antigen; RT: Room temperature; SD: Standard deviation.

## Competing interests

The authors declare that they have no competing interests.

## Authors’ contributions

BSD performed field work, supervised the laboratory work and wrote the initial draft. LG, AL, SS, GC and MM collected data, performed field and laboratory work and analysed data. ML collected data and performed field work. APS intellectually supported the study and corrected the drafts of the manuscript. All authors read and approved the final manuscript.
